# Supplementary material for: Emergency MRI in Spine Trauma of Children and Adolescents—A Pictorial Review
Source: Children (Basel). 2023 Jun 21;10(7):1094. doi: 10.3390/children10071094 (PMC10378627; doi:10.3390/children10071094)
Supplement: Supplementary file 1 [file children-10-01094-s001.zip › children-2460963-supplementary.pdf]

Table S1: Sequence parameters in emergency MRI of the pediatric spine

	Cervical spine					Thoracolumbar spine					Craniocervical junction		
Parameter	Sagittal T1	Sagittal T2	Sagittal STIR	Coronal STIR	Axial T2	Sagittal T1	Sagittal T2	Sagittal STIR	Coronal STIR	Axial T2	Sagittal PD	Coronal PD	Axial T2
TR (ms)	550	3584	3305	3721	5717	683	4955	4391	5778	3601	2500	2500	5902
TE (ms)	7	100	60	60	95	8	100	60	60	100	20	20	80
Flip angle	80	90	90	90	90	80	90	90	90	90	90	90	90
Slice thickness (mm)	3	3	3	3	3	3	3	3	4	3	2.5	2.5	2.5
Slice spacing	3.3	3.3	3.6	3.6	3.3	3.3	3.3	3.3	4.4	3.3	2.75	2.75	2.75
Number of slices	15	15	15	18	35	21	21	19	20	51	22	16	27
Matrix size	480 x 480	448 x 448	480 x 480	432 x 432	320 x 320	384 x 384	432 x 432	512 x 512	432 x 432	320 x 320	400 x 400	320 x 320	320 x 320
